# Antioxidant Metabolites in Primitive, Wild, and Cultivated Citrus and Their Role in Stress Tolerance

**DOI:** 10.3390/molecules26195801

**Published:** 2021-09-24

**Authors:** Muhammad Junaid Rao, Songguo Wu, Mingzheng Duan, Lingqiang Wang

**Affiliations:** 1 State Key Laboratory for Conservation and Utilization of Subtropical Agro-Bioresources, College of Agriculture, Guangxi University, 100 Daxue Rd., Nanning 530004, China; mjunaidrao@webmail.hzau.edu.cn (M.J.R.); wusongguo1128@163.com (S.W.); 2 Guangxi Key Laboratory of Sugarcane Biology, College of Agriculture, Guangxi University, 100 Daxue Rd., Nanning 530004, China; 3 Key Laboratory of Horticultural Plant Biology (Ministry of Education), Key Laboratory of Biology and Genetic Improvement of Horticultural Crops (Ministry of Agriculture), Institute of Citrus Science, Huazhong Agricultural University, Wuhan 430070, China

**Keywords:** citrus, antioxidant metabolites, flavonoids, stress tolerance

## Abstract

The genus *Citrus* contains a vast range of antioxidant metabolites, dietary metabolites, and antioxidant polyphenols that protect plants from unfavorable environmental conditions, enhance their tolerance to abiotic and biotic stresses, and possess multiple health-promoting effects in humans. This review summarizes various antioxidant metabolites such as organic acids, amino acids, alkaloids, fatty acids, carotenoids, ascorbic acid, tocopherols, terpenoids, hydroxycinnamic acids, flavonoids, and anthocyanins that are distributed in different citrus species. Among these antioxidant metabolites, flavonoids are abundantly present in primitive, wild, and cultivated citrus species and possess the highest antioxidant activity. We demonstrate that the primitive and wild citrus species (e.g., *Atalantia buxifolia* and *C. latipes*) have a high level of antioxidant metabolites and are tolerant to various abiotic and biotic stresses compared with cultivated citrus species (e.g., *C. sinensis* and *C. reticulata*). Additionally, we highlight the potential usage of citrus wastes (rag, seeds, fruit peels, etc.) and the health-promoting properties of citrus metabolites. Furthermore, we summarize the genes that are involved in the biosynthesis of antioxidant metabolites in different citrus species. We speculate that the genome-engineering technologies should be used to confirm the functions of candidate genes that are responsible for the accumulation of antioxidant metabolites, which will serve as an alternative tool to breed citrus cultivars with increased antioxidant metabolites.

## 1. Introduction

Citrus fruits are cultivated in more than 140 countries worldwide [1], of which China, Brazil, India, and the United States are major citrus-producing countries [1]; the global production statistics of citrus fruits are shown in Supplementary Figure S1. The *Citrus* genus belongs to the Rutaceae family that produces fruits of various sizes and shapes (from oblong to round). Some common citrus species are orange, mandarin, grapefruit, lemon, citron, and lime [2]. The citrus fruit provides abundant nutrition elements and dietary metabolites, including sugars, organic acids (e.g., citric acid), volatiles, amino acids, fibers, macro- and micro-nutrients, and vitamins B6, as well as an ample quantity of vitamin C [1,3]. Moreover, citrus fruits possess a variety of secondary metabolites such as alkaloids, limonoids, flavonoids, anthocyanins (color pigments), carotenoids, coumarins, phenol acids, and essential oils. These secondary metabolites have significant antioxidant and antimicrobial properties and are involved in UV photoprotection, internal regulation of plant cell physiology, reproduction, and signaling [4,5].

Consuming citrus fruits has been shown to have health-promoting effects on humans due to the antioxidant, anti-inflammatory, anti-cancer, cardiovascular protective, and neuroprotective properties of secondary metabolites in citrus fruits [6]. Moreover, citrus fruits are extensively used in the beverage, cosmetic, food, and pharmaceutical industries including medicines, spices, chemoprophylactic drugs, additives, etc. [7,8]. Also, the peels and fruits (mature and immature) of some citrus species, such as *C. reticulata* Blanco, *C. sinensis*, *C. medica* L., *C. wilsonii* Tanaka, and *C. saurantium* L., are widely used in traditional herbal medicine to cure cough, indigestion, muscle pain, ringworm infections, and skin inflammation, as well as to lower blood pressure [8,9].

Antioxidant compounds prevent, inhibit, or delay the process of oxidation [10,11]. Oxidation is a process by which free radicals are produced, thus leading to a series of chemical reactions that may directly or indirectly damage the cellular components (DNA, proteins, etc.) [12,13]. Citrus produces an ample quantity of endogenous antioxidants such as flavonoids, carotenoids, ascorbic acid (vitamin C), and tocopherols (vitamin E) that prevent the process of oxidation [12]. These antioxidants detoxify or reduce the negative effects of reactive oxygen species (ROS), thus protecting the cellular components from ROS damage. About two-thirds of the world’s plant species possess significant antioxidant potentials and show promising medicinal value [14].

Citrus species possess diverse and uneven levels of metabolites [12]. Some primitive and wild citrus species (e.g., *Atalantia buxifolia* and *C. latipes*) have high levels of metabolites (particularly phenolics and flavonoids) and are tolerant to various abiotic and biotic stresses, whereas the cultivated citrus species (e.g., *C. sinensis* and Cleopatra mandarin) containing less total metabolites are susceptible to abiotic and abiotic stresses [13,15]. The leaves, fruit juice, and phloem sap of cultivated citrus species showed a rapid increment in the antioxidant flavonoid and volatile compounds in response to biotic stress [16]. Some promising flavonoids were increased after abiotic or biotic stress including flavanone (e.g., hesperidin and naringenin), flavonol (e.g., quercetin), and some flavones [17]. These flavonoids have significant antioxidant and antimicrobial activities [18].

The genomic diversity and dissimilar levels of metabolites among *Citrus* species provide a promising opportunity to breed citrus cultivars with increased contents of metabolites [15,19]. However, the biosynthetic mechanisms of these antioxidant metabolites (e.g., flavonoids) in citrus are scarcely understood [1]. This review summarizes the genes that are involved in the biosynthesis of antioxidant metabolites, and their distribution in primitive, wild, and cultivated citrus. We deliberate the role of antioxidant metabolites in neutralizing ROS to enhance stress tolerance in citrus. Additionally, we highlight the antioxidant properties and therapeutic applications of citrus metabolites and discuss the potential usage of citrus wastes.

## 2. Importance of Antioxidants 

**Figure 1:**
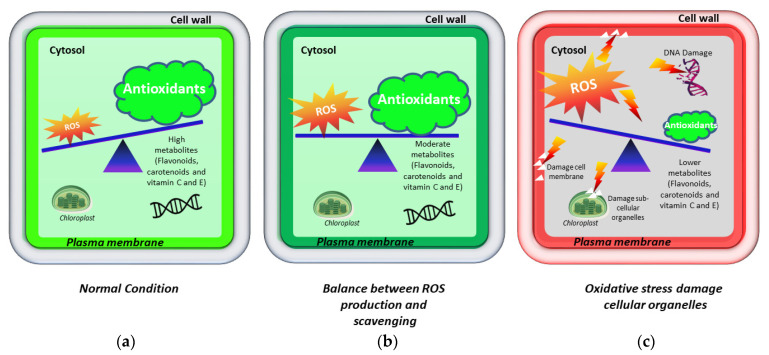
The role of antioxidant metabolites and reactive oxygen species (ROS) in normal and oxidative stress conditions. (**a**) High levels of antioxidant metabolites such as flavonoids can protect cellular organelles. (**b**) Under mild stress condition, the moderate level of antioxidant metabolites can detoxify ROS, which can maintain the balance between the production and scavenging of ROS. (**c**) Under high oxidative stress, the level of ROS is increased while that of antioxidant metabolites is reduced, which damages the membranes, DNA, proteins, and other cellular organelles, finally leading to cell death.

Antioxidants are chemical compounds that prevent, delay, or inhibit the process of oxidation of DNA, lipids, membranes, and proteins, thus directly protecting the cellular components from oxidative damage [20]. Antioxidants create a fine balance between the production and scavenging of ROS (Figure 1A–C). Plants have to face lower to serve degrees of oxidative stress during their life cycle and the availability of high antioxidant potential will be vital for their survival [21]. Generally, stress condition increases the rate of chemical reactions, thus triggering the production of free radicals (e.g., ROS) that cause serve oxidation of cellular components and eventually lead to cell death [10,21]. To overcome this situation, plants produce different kinds of metabolites that possess strong antioxidant activity, timely quenching free radicals and enabling crop plants to tolerate or acclimatize the stress conditions [10]. Trolox equivalent antioxidant capacity, 2,2-diphenyl-1-picrylhydrazyl, and ferric reducing antioxidant power are common antioxidant assays [22] that have been widely used to evaluate the antioxidant activity and capacity of citrus fruits [23].

The metabolic antioxidants have received a great deal of attention. Human beings also suffer from oxidative stress, which is a key causal factor of the progression and development of life-threatening sicknesses, including mental stress, muscle fatigue, and cardiovascular and neurodegenerative diseases [24]. Taken exogenous antioxidants from vegetables, fruits, citrus juice, etc. as supplementation of food will not only detoxify free radicals but also boost our body’s antioxidant defense system [25]. It has been proven to be a promising way to counteract the detrimental effects of ROS induced by oxidative stress.

## 3. The Producing Sites and Scavenging of ROS

**Figure 2:**
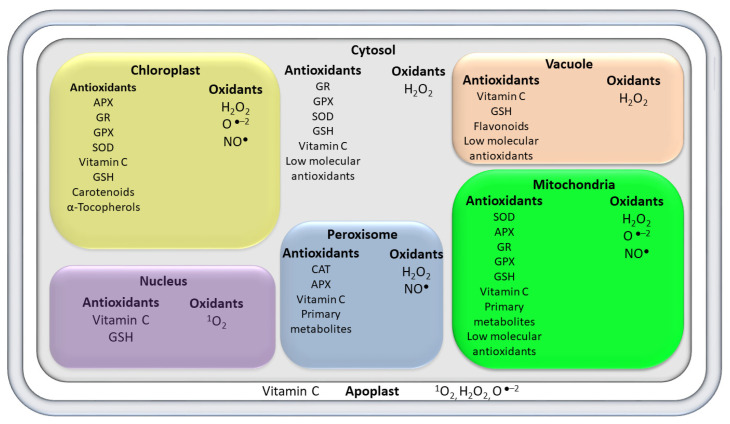
Distributions of antioxidants and oxidants in different subcellular organelles of the plant cells. APX, ascorbate peroxidase; GR, glutathione reductase; GPX, glutathione peroxidase; SOD, superoxide dismutase; CAT, catalase; GSH, glutathione; NO, nitric acid; ^1^O_2_, singlet oxygen; O•^−2^, superoxide; H_2_O_2_, hydrogen peroxide.

Mitochondria and chloroplasts are involved in maintaining an appropriate equilibrium among energy-linked functions and the production of ROS [26,27,28]. The matrix and membrane of the peroxisome, photosystems I and II (PS I and PS II) of the chloroplasts, ubiquinone, and complex I and complex III of the mitochondrial electron transport chain (ETC) are major ROS producing sites [29]. The peroxisomes produce nitric oxide (NO^●^), hydrogen peroxide (H_2_O_2_), and superoxide radicals (O_2_^●−^) (Table 1 and Figure 2); they also possess antioxidant enzymes such as catalase (CAT) and flavin oxidases [28]. Electron slippage occurs at PS I and PS II (in chloroplasts), the membrane of the peroxisome, and mitochondrial ETC (Table 1 and Figure 2). These electrons produce superoxide radicals (O_2_^−●^) by reacting with molecular oxygen, then O_2_^−●^ is consequently converted to hydroperoxyl radical (HO_2_^●^), and finally to H_2_O_2_ [10]. In addition, reactive nitrogen species (RNS) are the second type of free radicals that include peroxynitrite (ONOO-) and nitric oxide radical (NO^•^), which are also formed in different cellular organelles such as peroxisomes, chloroplasts, and mitochondria [30]. Moreover, reactive sulfur species (RSS) are the third type of free radicals that are generated by the reaction between thiols and ROS [29]. These free radicals are produced and quenched by antioxidants; however, the unfavorable environmental conditions can trigger the production of free radicals by distorting the normal cellular homeostasis (ROS production/scavenging balance) and cause serve damage to cellular biomolecules (Figure 1C).

Plants possess a variety of enzymatic and metabolic antioxidant defense mechanisms to reduce the harmful effects of free radicals [31]. The enzymatic antioxidant defense system includes superoxide dismutase (SOD), peroxidase (POD), CAT, glutathione peroxidase (GPX), ascorbate peroxidase (APX), glutathione reductase (GR), and glutathione S-transferases (GST) [10]. The metabolic antioxidant defense system includes low-molecular-weight molecules such as ascorbic acid, fatty acids, proline, carotenoids, amino acids, phenolic acids, flavonoids, and anthocyanins (color pigments), as well as high-molecular-weight secondary metabolites such as tannins [32]. These metabolic antioxidants are biosynthesized by plants due to two main reasons: firstly, the genetic makeup of plant species facilitates them to synthesize metabolic antioxidants; secondly, the biosynthesis of antioxidant metabolites enables plants to respond to unfavorable environmental conditions [32]. The biological activities of enzymatic and metabolic antioxidants and their reaction with ROS are summarized in Table 1.

## 4. Diversity of Antioxidant Metabolites in *Citrus*

Citrus species contain a variety of antioxidant metabolites, which are divided into primary (e.g., fatty acids, amino acids, and organic acids) and secondary metabolites (e.g., phenolics, flavonoids, carotenoids, limonoids, and alkaloids) (Table 1 and Figure 2). The details of each class of citrus metabolites are discussed below.

### 4.1. Antioxidant Volatiles and Fatty Acids

Volatile compounds possess moderate to high antioxidant activities in citrus plants [33]. Most of the volatile compounds are extracted from the citrus fruit peels and they possess antioxidant, antimicrobial, antioxidative, and cytotoxic properties [34]. Some common volatile compounds reported in different citrus species are α-pinene, β-pinene, sabinene, myrcene, p-cymene, α-terpinene, terpinolene, linalool, neryl acetate, geranyl acetate, caryophyllene, terpinene-4-ol, β-elemene, neral, nerol, α-farnesene, β-farnesene, α-terpineol, geraniol, thujene, α-phellandrene, β-phellandrene, octanal, limonene, decanal, citronellal, heptanal, nonanal, valencene, ethyl heptanoate, geranyl acetone, hexyl acetate, ethyl nonanoate, ethyl octanoate, undecanal, citronellol, ethanol, styrene, geranial, thymol, sativene, β-santalene, and β-selinene [35,36,37,38].

Citric acid and malic acid are commonly found in citrus fruits, whereas oxalic acid, tartaric acid, benzoic acid, succinic acid, and malonic acid are present in traces [39]. α-Linolenic acid (an essential fatty acid) and α-lipoic acid (a well-known antioxidant) are two key molecules biosynthesized by citrus plants. α-Lipoic acid helps to neutralize free radicals, and α-linolenic acid is the precursor of many lipids and is essential for good health. Furthermore, organic acids are present in different citrus species such as orange, mandarin, lemon, lime, grapefruit, and tangerine. Organic acids have moderate antioxidant activity and are very useful due to their bioactivity and sensory properties [40].

### 4.2. Antioxidant Alkaloids, Coumarins, and Limonoids

Bioactive alkaloids are abundantly present in different citrus species and possess significant antioxidant activities. Alkaloids are indirectly involved in the growth, reproduction, and metabolism of citrus plants [41,42], of which *C. aurantium* contains higher levels of antioxidant alkaloids than other *Citrus* species [43]. Some commonly reported alkaloids in citrus plants are (±)octopamine, tyramine, *N*-methyltyramine, hordenine, *N*-methylnicotinic acid, and (±)synephrine [41,42]. Synephrine is most dominant among the alkaloids found in citrus species. The synephrine alkaloid is present in more than 85% of the total protoalkaloid content found in citrus [43]. Also, higher levels of *N*-methyltyramine compound have been observed in citrus species than those of hordenine, octopamine, and tyramine [42].

High concentrations of coumarins are usually found in the peels of *Citrus* species [44]. Coumarin compounds such as transferrin, limettin, auraptene, isomeranzin, umbelliferone, herniarin, psoralen, bergamottin, ecxybergamottin, 5-hydroxyfurocoumarin, bergapten, osthol, and 8-geranyloxypsoralen have been reported in different *Citrus* species [45]. Among various groups of coumarins, auraptene (7-geranyloxycoumarin) is the key coumarin that is found plentifully in *Citrus* species. Coumarins from *Citrus* species have shown anti-inflammatory, antibacterial, and antioxidant activities [44]. Previous studies have suggested that di-hydroxy-coumarins possess better antioxidant activity compared with mono-hydroxy-coumarins [46]. In the coumarin skeleton, the position of OH groups near C6 and C7 plays an important role in the bioactivities of coumarins [47].

Limonoids are mainly found in the forms of glucosides, A-ring lactones, and aglycones [48]. Limonoids are highly oxygenated triterpenoids that are precursors of limonoid glucosides and aglycones [49]. Limonin (from the limonoid group) is prominently found in the Meliaceae and Rutaceae families [48]. *Citrus* species also contain several limonoid compounds such as limonin, nomilin, obacunone, obacunone acetate, deacetyl-nomilin, deoxylimonin, methyldeacetylnomilinate, ichangin, ichangensin, calamine, nomilin glucoside, limonin glucoside, nomilinic acid glucoside, and citriolide-A [50]. Among different detected limonoids, limonin and limonin glucoside are measured in high concentrations in *Citrus* species [49]. Some limonoids showed better antioxidant activities than vitamin C. Limonin, obacunone, deacetylation millington acid, and millington acid are four limonin glycosides that possess the strongest free radical quenching activity compared with other limonoids. The millington acid shows resilient free radical scavenging activity whereas limonin exhibits the lowest antioxidant activity [51]. The genes involved in the biosynthesis of different metabolites in citrus are documented in Table 2.

### 4.3. Antioxidant Carotenoids, Ascorbic Acid, and Tocopherols in Citrus

Carotenoids are isoprenoid-derived biomolecules that are characterized as lipophilic antioxidants. Carotenoids are abundantly synthesized by plants and are divided into two sub-groups: carotenes (contain carbon and hydrogen atoms) and xanthophylls (oxygenated forms of carotenes) [52]. The carotenoid pigments are randomly distributed in various vegetables and orange-colored fruits such as citrus, apricot, carrot, spinach, mango, sweet potato, papaya, and squash. In plants, the biosynthetic pathway of carotenoids is phytoene → phytofluene → ζ-carotene → neurosporene → lycopene, and lycopene is then converted to α-carotene and β-carotene [12]. Carotenoids exhibit significant antioxidant activities and can detoxify/quench considerable amounts of peroxyl radicals and singlet molecular oxygen [52].

Carotenoids are abundantly found in *Citrus* species and endow yellow to orange color to citrus fruits. However, the carotenoid concentration is tissue-specific and varies from species to species [12]. Many kinds of carotenoids (e.g., violaxanthin, β-cryptoxanthin, α-carotene, lutein, lycopene, zeaxanthin, antheraxanthin, cryptoxanthin, phytoene, phytofluene, β-citraurin, β-Carotene, and neoxanthin) have been reported in different *Citrus* species including *C. aurantifolia, C. aurantium, C. clementina, C. grandis, C. hystrix, C. limon, C. limonimedica, C. medica, C. reticulata*, and *C. sinensis* [53]. Of these carotenoids, cryptoxanthin, β-carotene, α-carotene, and zeaxanthin are active quenchers of ROS (particularly singlet molecular oxygen) [52]. Due to higher antioxidant activities, these carotenoids not only protect plants from abiotic and biotic factors but also prevent humans from a wide range of chronic diseases [54]. β-Carotene and lycopene are considered provitamin A carotenoids. In humans, the bioavailability of provitamin A has been extensively studied and our body converts this provitamin A compound into retinol, which is an active form of vitamin A [54]. These carotenoids harbor a variety of functions in plants such as protecting plant cells from oxidative damage during photosynthesis, interacting with pathogens and pests, serving as the substrate of hormones, endowing plants with different colors to attract pollinators, being involved in seed dispersal, and participating in plant cross-talk with symbiotic organisms [12,54]. Recently, some genes were identified to govern carotenoid biosynthesis in different *Citrus* species (Table 2).

Ascorbic acid is a powerful water-soluble antioxidant that is synthesized in the mitochondrion and then transported through facilitated diffusion or by a proton-electrochemical gradient to nearby subcellular organelles [29]. Ascorbic acid is the strongest antioxidant molecule because it can donate electrons to a variety of non-enzymatic and enzymatic reactions [55]. Ascorbic acid directly quenches the OH• and O_2_•^−^ ions and is involved in the regeneration of oxidized α-tocopherol or carotenoids, thus reducing the damage caused by the oxidative process (through synergic action by other antioxidants) and providing protection to the membrane [29,55]. Plant cells maintain a high level of ascorbic acid via a proficient recycling system, which makes ascorbic acid an appropriate antioxidant [56]. Almost all *Citrus* species have high levels of ascorbic acid, but the levels vary among different plant tissues; moreover, high contents of ascorbic acid are found in citrus fruits juice, photosynthetic cells, and meristems [57]. A high level of ascorbate has been found in the cytosol, while plastids and the mitochondrion have moderate ascorbate levels and vacuoles have the lowest ascorbate levels.

Tocopherols are lipid-soluble antioxidants that are synthesized by all plants. Tocopherols protect cellular components and lipids by quenching and scavenging several lipid by-products and ROS [69]. In plants, tocopherols have four isomers; one of them is α-tocopherol, a key antioxidant that represents vitamin E and is located in the thylakoid membrane and chloroplast envelope [70]. α-Tocopherol has the highest antioxidant activity and is also involved in membrane rigidity. Previous studies reported that tocopherol concentrations were increased significantly after water and chilling stresses [69,71], and tocopherol-deficient plants exhibiting irregular cellular signaling were more prone to oxidative stress. In addition, tocopherols have significant health-promoting effects on the human body due to their antioxidant activities [70,71].

### 4.4. Antioxidant Amino Acids

*Citrus* varieties tolerant to Huanglongbing (HLB) disease, such as *C. latipes* [72] and orange jasmine (*Murraya paniculata*) [73], are higher in total antioxidant amino acids. High levels of antioxidant amino acids protect plant cells from the negative effects of ROS [73]. *Citrus* varieties tolerant to HLB (e.g., *A. buxifolia* and *M. paniculata*) possess considerable amounts of amino acids such as valine, serine, aspartic acid, threonine, asparagine, and proline [72]. Particularly, anthranilic acid and gamma-amino-butyric acid is specifically higher in *C. latipes*. Furthermore, some semi-tolerant *Citrus* varieties, such as Volkamer lemon and Palestine sweet lime, harbor higher levels of amino acids such as asparagine, phenylalanine, arginine, and threonine; these amino acids are famous due to their antioxidant potential and can protect plant cells from ROS damage [73,74]. Generally, the primitive (*A. buxifolia*) and wild citrus (*C. latipes*) species possess higher levels of antioxidant amino acids compared with the cultivated citrus species (*C. sinensis*) [72]. The tolerant citrus germplasms possess a high amount of amino acids, particularly those having high antioxidant potential such as lysine, tyrosine, phenylalanine, tryptophan, and asparagine. Moreover, these amino acids have been demonstrated to be associated with plant defense against several abiotic and biotic stresses [73]. Interestingly, the cultivated *Citrus* species such as *C. sinensis* and *C. reticulata* have lower levels of antioxidant amino acids; however, they biosynthesize antioxidant amino acids (e.g., lysine, tyrosine, phenylalanine, and tryptophan) under abiotic or biotic stress, indicating these amino acids may contribute to stress resistance of cultivated *Citrus* species [73]. The average amount of bioactive metabolites and antioxidant capacity of different citrus varieties are presented in Table 3.

In *Citrus* species, higher levels of antioxidant amino acids such as phenylalanine, tyrosine, and tryptophan are positively associated with stress tolerance [72]. Most of the secondary metabolites and derivatives of hydroxycinnamic acids (phenolic compounds) such as flavonoids are derived from phenylalanine, tyrosine, and tryptophan [75]. The higher levels of phenylalanine, tyrosine, and tryptophan will facilitate the rapid biosynthesis of phenolic compounds under any unfavorable environmental conditions [73]. Prompt endogenous biosynthesis of phenolic compounds in the least time after pathogen invasion is supposed to be more important than their endogenous concentrations in plants [16]. The plant species that possess a high level of antioxidant phenolic compounds are least attractive to pathogens [75,76]. To conclude, the primitive and wild citrus species have a high concentration of total amino acids, and they biosynthesize antioxidant phenolic compounds (such as flavonoids) more rapidly than cultivated *Citrus* species.

### 4.5. Hydroxycinnamic Acids and Their Derivatives

Hydroxycinnamic acids (HCAs) are commonly found in all *Citrus* species, which give rise to a diverse class of secondary metabolites [79]. Some key HCAs are randomly found in *Citrus* species, such as sinapic acid, p-coumaric acid, ferulic acid, caffeic acid, trans-2-hydroxycinnamic acid, trans-cinnamic acid, rosmarinic acid, protocatechuic acid, p-hydroxybenzoic, vanillic acid, gallic acid, chlorogenic acid, ferulic-*O*-hexoside, sinapic-*O*-hexoside, and syringic acid [80]. Previous metabolic studies on *Citrus* species showed that four structurally related HCAs significantly accumulated in the leaves of *C. sinensis* exposed to abiotic and biotic stresses [81]. HCAs and their derivatives such as p-coumaric acid, ferulic acid, and caffeic acid possess significant antioxidant activities and can detoxify the ROS produced during oxidative stress [13]. Besides, HCAs have strong antimicrobial activities. For example, the HCA levels were significantly increased in cucumber after *Prunus* necrotic ringspot virus invasion and powdery mildew infestation [82], as well as in tomato after bacterial (*Pseudomonas syringae*) attack [83].

### 4.6. Antioxidant Flavonoids

Flavonoids are one of the major classes of secondary metabolites, which are extensively found in citrus fruit peel, fruit juice, leaves, and roots [84,85]. Among the secondary metabolites, flavonoids possess the highest antioxidant, antimicrobial, antiallergy, anti-inflammatory, and anticancer activities. In addition, flavonoids have cardiovascular, hepatoprotective, and neuroprotective effects and are used for obesity control [80]. Flavonoids are further divided into different subclasses including flavanone, flavone, flavanonol, flavonols, isoflavones, and anthocyanins [80]. Several subclasses of antioxidant flavonoids have been isolated from different *Citrus* species (including *C. aurantifolia*, *C. aurantium*, *C. clementina*, *C. grandis, C. unshiu, C. hystrix, C. limon, C. paradisi, C. limonimedica, C. medica, C. reticulata*, and *C. sinensis*) and are characterized as flavanone and flavanonol: naringenin, hesperetin, narirutin, naringin, hesperidin, neohesperidin, eriocitrin, neoeriocitrin, poncirin, and didymin [86]; flavone and flavonol: luteolin, apigenin, quercetin, luteolin-6,8-di-C-glucoside, luteolin-7-*O*-rutinoside, apigenin-6,8-di-C-glucoside, apigenin-7-*O*-rutinoside, diosmin, rutin, chrysoeriol, chrysoeriol-7-*O*-rutinoside, chrysoeriol-6,8-di-C-glucoside, quercetin, quercetin-7-*O*-rutinoside, kaempferol, and kaempferol-3-*O*-rutinoside [87]; poly-methoxylated-flavones: sinensetin, nobiletin, tangeretin, isosinensetin, 3,5,6,7,8,3′,4′-heptamethoxyflavone, and 5,7,8,3′,4′-pentamethoxyflavone [88,89]; and color pigments: proanthocyanidins and anthocyanins [84]. Among different flavonoid subclasses, flavanone is abundantly found in *Citrus* species with the highest antioxidant activity compared with other flavonoids [90]. Some key bioactive flavonoids such as naringenin, naringin, hesperetin, hesperidin (flavanone), tangeritin, and nobiletin (polymethoxylatedflavone) are extensively studied and are not only resilient antioxidants, but also have antimicrobial, anticancer, and anti-inflammatory properties [90].

**Figure 3:**
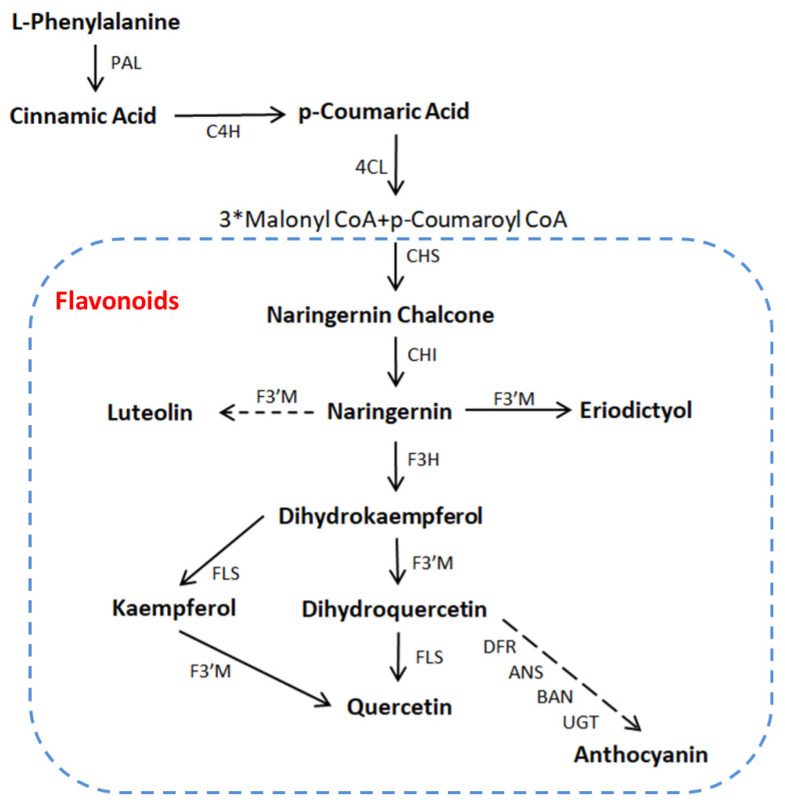
Flavonoid biosynthesis pathway. Gene abbreviations were taken from KEGG (www.genome.jp/kegg/pathway accessed on 14 August 2021) for plants. PAL, phenylalanine ammonia lyase; C4H, cinnamate 4-hydroxylase; 4CL, 4-coumarate: CoA ligase; CHS, chalcone synthase; CHI, chalcone isomerase; F3H, flavanone 3-hydroxylase; F3′M, flavonoid 3′-monooxygenase; FLS, flavonol synthase; DFR, dihydroflavonol 4-reductase; ANS, anthocyanidin synthase; BAN, banyuls; UGT, UDP-glucosyl transferase 78D3.

**Figure 4:**
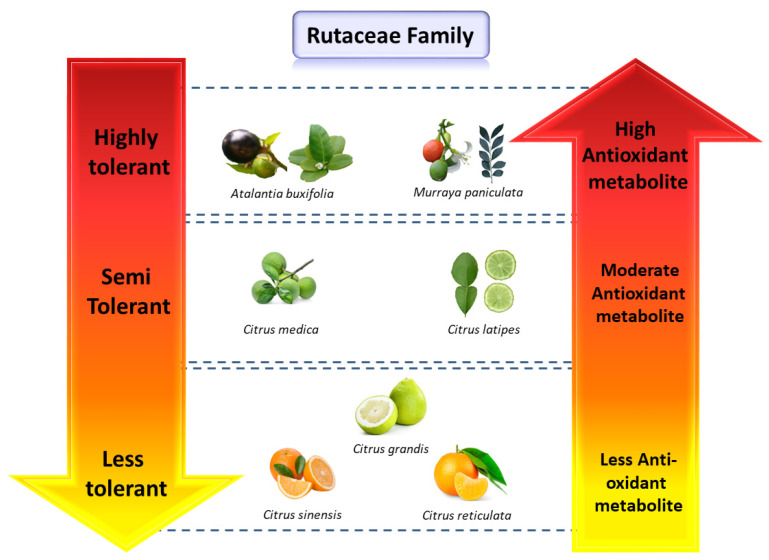
Correlation among antioxidant metabolites and stress tolerance in different *Citrus* species. The primitive citrus species that have a high level of metabolites are tolerant to various abiotic and biotic stresses; the wild citrus species (*C. medica* and *C. latipes*) possessing moderate levels of metabolites are semi-tolerant to stresses; and the cultivated citrus species that contain fewer metabolites are more prone to stresses. High levels of metabolites are positively correlated with abiotic [13] and biotic stress tolerance in citrus [15].

Flavonoids are localized in plant vacuoles and are considered the most powerful antioxidant compounds [84]. The relationship between flavonoid activity and ROS scavengers has been determined [84,85]. The catechol structure in the B ring (heterocyclic ring) is the key contributing factor for the scavenging activities of flavonoids [86]. Flavonoids are involved in the processes of plant resistance against pathogens, pollination attraction, and seed dispersal facilitation; possess antibacterial, antifungal, and antiviral activities; and can scavenge ROS and defend against insects and pests [91,92]. Flavonoid biosynthesis is increased significantly after serve abiotic (metal toxicity, drought, wounding, high-light stress, chilling, salt stress, radiation, and nutrient deficiency [19,65,84]) and biotic stresses (e.g., bacterial, fungal, and viral infection) in *Citrus* species [15]. The genes involved in the biosynthesis of antioxidant flavonoids are demonstrated in Figure 3. In citrus, the concentration of antioxidant metabolites is positively correlated with stress tolerance (Figure 4).

Pummelo, sweet orange, and mandarin are very close to each other metabolically (Table 3 and Figure 4); the genomic data of these species also showed similar results (Supplementary Figure S2). It was revealed that sweet orange is derived from the interspecific hybridization between mandarin (male parent) and pummelo (female parent) followed by backcrossing with mandarin (male parent), i.e., sweet orange = (pummelo × mandarin) × mandarin [93]. The genetic relationship among sweet orange, pummelo, and mandarin results in a close metabolic correlation. The variation in the genetic makeup might be the main reason for the dissimilar distribution of metabolites among primitive, wild, and cultivated citrus species. In the future, editing or cloning of promising genes from primitive and wild citrus species (that are responsible for metabolic synthesis) and overexpressing them in cultivated citrus will be a novel strategy to improve the endogenous metabolic potential of cultivated citrus species. A high level of metabolites will not only increase the tolerance of citrus, but can also help to overwhelm the nutritional deficiency in humans.

## 5. Therapeutic Applications of Citrus Fruits and Potential Usage of Citrus Wastes

Citrus fruits provide an impressive list of phytochemicals, nutrients, antioxidants, and bioactive chemicals that are required for a balanced diet and can prevent humans from various diseases such as inflammation, heart diseases, gastrointestinal diseases, cancers, tumors, and obesity [94,95,96,97]. Flavonoid compounds that are abundantly present in citrus fruits such as nobiletin, neohesperetin, hesperetin, and tangeretin have significant tumor-suppressing properties in the human body [98]. For example, d-limonene (in citrus peel oil) shows significant anticancer activity; using citrus peel oil is effective in controlling skin cancer since d-limonene can suppress tumor cell growth [99]. Extracts from *C. sphaerocarpa* (Korean hallabong) peels present inhibitory effects on breast cancer metastasis [100].

All commercially cultivated citrus species contain an abundant amount of ascorbic acid (a powerful antioxidant agent) that is considered an immune booster and prevents humans from a variety of chronic and infectious diseases, muscle fatigue, and oxidative damage [94]. Also, citrus fruits have plentiful hesperidin (a flavonoid), which can help humans fight against the novel SARS-CoV-2 coronavirus (COVID-19) [101]. Tyrosine has been shown to have good anticancer activity. Citrus fruit juice displays antioxidant and antiproliferative effects (tending to inhibit cell growth) on different patients suffering from cancer [100]. Generally, taking an unnecessary and imbalanced diet will lead to bowel diseases and colon cancers, and about 90% of colon cancers worldwide are caused by improper diet habits [102]. Polyphenols and enzymes from citrus fruit juice (e.g., grapefruit juice) can control and treat cancers as well as obesity of the human body [103]. Moreover, citrus fruits possess enormous kinds of bioactive compounds (e.g., nobiletin, hesperidin, and flavones) that play an extraordinary role in hepatic mechanisms (distortion of the vascular architecture and liver parenchyma); specifically, sweet orange and lemon are reported to be able to control 60–70% of liver diseases [104].

Citrus fruits are composed of juice (45%), rag and pulp (26%), inner peel albedo (17%), outer peel flavedo (10%), and seeds (2%) [105]. The pulp and juice are edible, whereas the peel and seeds are non-edible or waste production of citrus fruits [105]. The waste materials of citrus fruits (such as seeds, segment wall, flavedo, albedo, rag, and pith residue) provide renewable sources for the production of valuable compounds that are widely used in cosmetic, food, nutraceutical, and pharmaceutical industries [105,106]. The citrus fruit peel is a good source of polymethoxyflavonoids, flavonoids, saponins, phenolic compounds, essential oils, and tannins [6], which have immunosuppressive, hepatoprotective, and antimicrobial effects (against dental caries bacteria *Lactobacillus acidophilus* and *Streptococcus mutans*) [107]. Moreover, the polyphenolic compounds possess an inhibitory effect on breast cancer metastasis, a cytotoxic effect on colorectal carcinoma cells, and antioxidant and antiulcer activities in humans [33,100,105,106]. The citrus albedo provides abundant dietary fiber, reducing the risk of cancers [105]. Essential oils from the *C. limon* peel revealed a resilient antifungal effect on oral candidiasis fungus (*Candida albicans*) because the *C. limon* peel contains terpenoids that prevent ergosterol synthesis and destroy the fungal cell wall (extinguish cell membrane permeability) [108]. Also, citrus wastes contain complex polysaccharide content and coloring material, which are widely used by soft drink/beverage industries as clouding agents [109]. Moreover, the citrus segment membrane, peel, and other by-products are dried and used as raw material to extract pelletization or pectin in animal feed [110].

## 6. Genomic Features of *Citrus* Species

The complex and diverse genomic features of citrus species lead to the uneven distribution of metabolites among *Citrus* species. The intraspecific variation and heterozygosity in some citrus species (e.g., sweet orange and some mandarins) and interspecific admixture (a mechanism involving complex backcrosses), origins for enormous variations at the genomic level, both result in significant dissimilarities in the biosynthesis of metabolites [111]. Some pure citrus genotypes (e.g., citrons without interspecific admixture) exhibit considerably reduced intraspecific diversity (about 0.1%) than other *Citrus* species. In addition, some primitive citrus species (e.g., *A. buxifolia*) are sexually propagated (assists natural variation at the genomic level), whereas the cultivated citrus species such as sweet orange and mandarin are asexually propagated thus reducing the uncertainty associated with sexual reproduction [93,112]. Therefore, inducing any trait of interest such as enhancing antioxidant metabolites in cultivated citrus species will require genome engineering tools. The difference and variations in the genomic features between primitive and cultivated citrus will offer a unique opportunity to induce or edit the genes associated with high production of antioxidant metabolites.

In the past few years, the rapid success in the field of genome editing and the invention of clustered regularly interspaced short palindromic repeat (CRISPR) genome engineering technology have revolutionized the field of molecular and genetic research [113]. The use of CRISPR to knock-in the desired gene of interest to enhance the metabolites or knock-out the candidate gene to trigger endogenous production of the desired antioxidant flavonoids/metabolites will be a novel strategy to boost antioxidant potential in citrus plants. Interestingly, the publicly available genomes of nine citrus species, including primitive, wild, and cultivated species [112,114,115], provide a novel opportunity to understand the biosynthetic mechanism of these metabolites and to breed citrus cultivars with increased endogenous metabolic antioxidants.

## 7. Conclusions

We conclude that citrus fruits are an ample source of antioxidant metabolites such as volatiles, fatty acids, alkaloids, coumarins, limonoids, carotenoids, ascorbic acid, tocopherols, terpenoids, amino acids, hydroxycinnamic acids, and flavonoids. In this review, we highlight that the primitive and wild citrus species, having high levels of antioxidant metabolites, are more tolerant to abiotic and biotic stresses compared to cultivated citrus species. Additionally, we abridge promising genes that are involved in the biosynthesis of antioxidant metabolites and their role in stress tolerance. Furthermore, we discuss the potential usage of citrus wastes and the therapeutic application of citrus metabolites. In the future, genome-editing technologies should be used to unravel the biosynthetic mechanism and regulatory pathways of antioxidant metabolites (i.e., flavonoids) to trigger the endogenous synthesis of flavonoids, which will ultimately enhance stress tolerance in cultivated citrus varieties.

## Figures and Tables

**Table 1:** Chemical reaction of enzymatic and metabolic antioxidants with reactive oxygen species (ROS).

ROS	Reacts with	Enzymatic Scavenging System	Metabolic Antioxidants	Reaction with ROS to Enhance Stress Tolerance
Superoxide (O•^−2^)	Fe–S proteins dismutate to H_2_O_2_	SOD EC 1.15.1.1	Proline/Glycine betaine	Helps in enhancing stress tolerance
Hydrogen peroxide (H_2_O_2_)	Proteins, heme-proteins, and DNA	CAT EC 1.11.1.6 GPX EC 1.11.1.9 GST EC 2.5.1.18 APX EC 1.11.1.11	Amino acids, carotenoids, α-tocopherol/ascorbic acid, and glutathione	Hunts ROS
Singlet oxygen (^1^O_2_)	Oxidized lipids, G-residues of DNA, and proteins	-	Carotenoids and α-tocopherol (vitamin E)	Neutralizes free radicals and protects the photosynthetic apparatus from ROS
Hydroxyl radical (OH•)	DNA, RNA, lipids, and proteins	-	Flavonoids, sugars, proline Ascorbate.	Helps in maintaining cell homeostasis
Other reactive radicals	-	POD EC 1.11.1.x GR EC 1.6.4.2	Fatty acids/organic acids and polyphenols (flavonoids)	Protect cells from negative effects of ROS by trapping free radicals

APX, ascorbate peroxidase; SOD, superoxide dismutase; GPX, glutathione peroxidase; GST, glutathione S-transferases; POD, peroxidase; GR, glutathione reductase; CAT, catalase.

**Table 2:** Genes involved in the biosynthesis of different metabolites in *Citrus* species.

Serial No.	Genes	Identified in	Common Name	Category	Metabolism	Involved in	References
1	*CrMYB68*	*Citrus reticulata* cv. Suavissima	Mandarin	R2R3-MYB transcription factor	Carotenoid metabolism	α- and β-branch carotenoids	[58]
2	*UGT708G1*	*Fortunella crassifolia*	Kumquat	UGT-glucosyltransferase enzyme	Flavonoid accumulation	Anthocyanin biosynthesis	[59]
3	*UGT708G2*	*Citrus unshiu*	Satsuma mandarin	UGT-glucosyltransferase enzyme	flavonoid accumulation	Anthocyanin pigments	[59]
4	*CgMYB58*	*Citrus maxima*	Pummelo	MYB transcription factor	Lignin biosynthesis	Lignin accumulation in juice vesicles	[60]
6	Ruby and Noemi (bHLH)	*Citrus sinensis, Citrus medica*, and their hybrid	Orange, citron, and their hybrid	Transcription factor	Color formation	Flavonoid and anthocyanin biosynthesis	[61]
7	*CsMYB3* and *CsRuby1*	*Citrus sinensis*	Sweet orange	Transcription factor	Anthocyanin biosynthesis	Anthocyanin pigment accumulation	[62]
8	*CCD4*	*Citrus reticulata*	Mandarin and its hybrids	CAROTENOID CLEAVAGE DIOXYGENASE	Carotenoid metabolism		[63]
9	*CsMADS6*	*Citrus sinensis*	Sweet orange	Transcription factor	Carotenoid metabolism	Activating downstream carotenoid genes	[64]
10	*CsUGT78D3*	*Citrus sinensis*	Sweet orange	UDP-glucosyl transferase enzyme	Enhances proanthocyanidins and anthocyanins	High light stress tolerance by high anthocyanin contents	[65]
11	*CsCYT75B1*	*Citrus sinensis*	Sweet orange	Cytochrome P450 75B1 enzyme	Flavonoid biosynthesis	Drought tolerance due to high flavonoid content	[19]
12	*CWINVs, VINV, SPS2, SUT2, VPPs*	*Citrus sinensis* (HAL)	Orange (Hong Anliu)	Genes encoding enzymes	Sugar	Sugar accumulation	[66]
13	*CitLGT*	*Citrus unshiu* Marc.	Satsuma mandarin	Limonoids UDP-glucosyl transferase enzyme	Limonoid GTase	Converting limonoid aglycones to glucosides	[67]
14	*CpGTs*	*Citrus paradisi* cv. Duncan	Grapefruit	Glucosyltransferases (GTs)	Color development	Color development	[68]

**Table 3:** The average amount of bioactive compounds and antioxidant capacity of different *Citrus* species [23,73,77,78].

Citrus Species Common and Scientific Name	Antioxidant Capacity (µmol TE/100 g)	Total Phenolics (mg Gallic Acid Equivalent/g)	Total Amino Acids (g/100 g of Sample)	Total Carotenoids (mg/kg)	Total Flavonoids (mg/100 mL Juice)	Total Volatiles (1 Unit Equals to 10 mg/g Fresh Weight)	β-Carotene (mg/kg)	Lycopene (mg/kg)	Ascorbic Acid (mg/kg)	Total Acidity (g/100 mL Juice)
Robinson (*Citrus reticulata*)	20.45 ± 0.98	209.37 ± 1.37	-	26.67 ± 0.67	-	-	22.67 ± 0.54	4.19 ± 0.12	651.33 ± 0.93	0.563
Clementine (*Citrus reticulata*)	33.10 ± 0.68	302.38 ± 0.91	25.54 ± 0.22	27.23 ± 0.12	19.23 ± 0.97	191.23 ± 1.29	22.33 ± 0.13	3.27 ± 0.20	656.43 ± 1.03	0.588
Cocktail (*Citrus paradisi*)	45.28 ± 0.76	214.88 ± 0.87	-	37.40 ± 0.33	-	155.4 ± 2.51	31.79 ± 0.93	3.20 ± 0.07	353.17 ± 0.77	
Valencia (*Citrus sinensis*)	40.32 ± 1.01	270.56 ± 0.67	20.56 ± 0.31	29.87 ± 0.98	18.34 ± 1.22	289.43 ± 4.81	25.89 ± 0.36	2.09 ± 0.24	579.99 ± 1.10	1.024
Wild lime (*Citrus hystrix*)	83.91 ± 0.81	490.74 ± 1.75	-	-	22.25 ± 0.20	-	-	-	-	-
Common lime (*Citrus aurantifolia*)	69.54 ± 0.58	211.70 ± 0.0	-	-	10.67 ± 0.27	512.92 ± 2.19	-	-	-	3.328
*Citrus maxima*	29.34 ± 1.04	501.43 ± 2.98	23.9 ± 0.9	23.17 ± 1.67	19.45 ± 0.65	-	-	-	-	-
*Atalantia buxifolia*	74.24 ± 2.19	645.89 ± 3.47	24.84 ± 0.16	27.83 ± 1.45	28.53 ± 1.24	1567.11 ± 3.82	-	-	-	-
*Poncirus trifoliata*	-	-	-	-	-	145. 78 ± 1.35	-	-	-	-

## Data Availability

Not applicable.

## Supplementary Materials

The following are available online, Figure S1: The citrus-producing countries around the world (FAO 2017). Figure S2: Phylogenetic tree of citrus germplasms constructed based on published genomic data by RAxML and visualized by iTOL.
