# Supplementary material for: Antioxidant Metabolites in Primitive, Wild, and Cultivated Citrus and Their Role in Stress Tolerance
Source: Molecules. 2021 Sep 24;26(19):5801. doi: 10.3390/molecules26195801 (PMC8510114; doi:10.3390/molecules26195801)
Supplement: Supplementary file 1 [file molecules-26-05801-s001.zip › molecules-1363294-supplementary.pdf]

The Figure S1 is representing the world's map showing the production scenario of citrus in the world (FAO 2017). So, China, Brazil, India, United States and the region surrounding these countries produces the highest quantity of citrus (Figure 1).

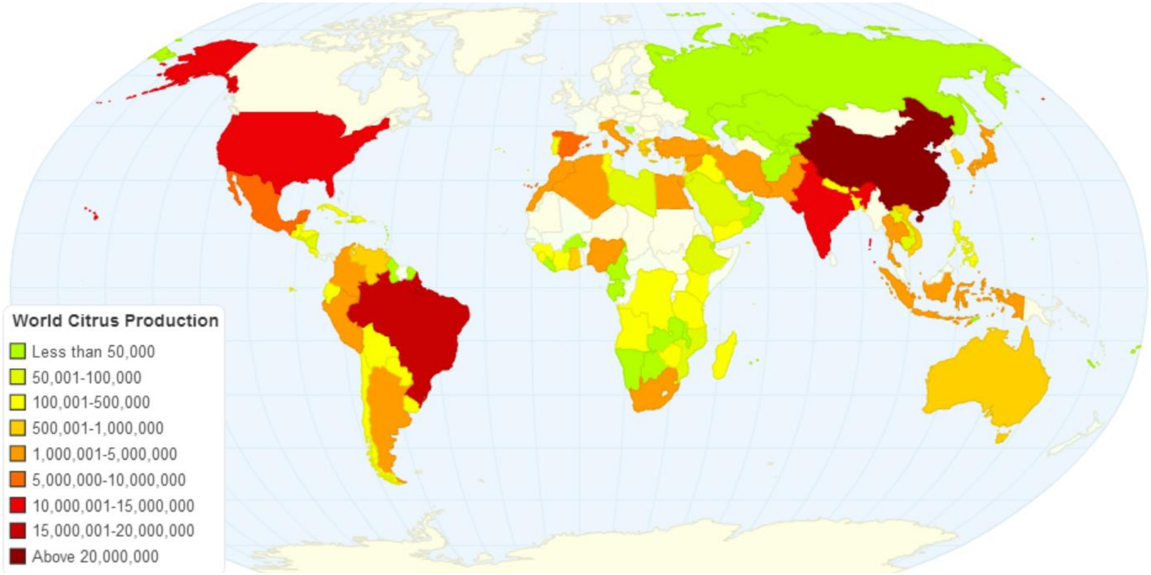


**Figure S1.** The citrus-producing countries around the world (FAO 2017). (Internet source: http://chartsbin.com/view/37266).

According to Food and agriculture organization (FAO) statistical data of 2017 the world's citrus production was 124,246.0 thousand tons (TT) and China stands first with highest production about 32,705.9 TT and shares 26.3% of world's total citrus produced followed by Brazil.

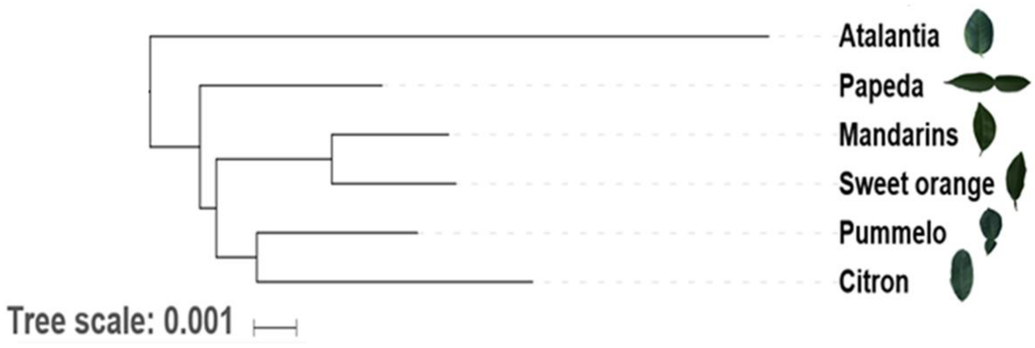


**Figure S2.** Phylogenetic tree of citrus germplasms constructed based on published genomic data by RAxML and visualized by iTOL.

## References

1. FAO. Statistical Database (FAOSTAT), Citrus fruit-fresh and processed statistical bulletin. *Food and Agriculture Organization of the United Nations*, 2017, http://www.fao.org/statistics/en/.
